# Expansion of The ABCA4-Associated Retinopathy Spectrum: Might Vitamin A Supplementation Have Contributed to a More Severe Phenotype?

**DOI:** 10.1167/iovs.66.11.41

**Published:** 2025-08-18

**Authors:** Omar A. Mahroo

**Affiliations:** 1 Institute of Ophthalmology, University College London, London, United Kingdom; 2 NIHR Biomedical Research Centre at Moorfields Eye Hospital and the UCL Institute of Ophthalmology, London, United Kingdom; 3 Genetics Service, Moorfields Eye Hospital, London, United Kingdom; 4 Section of Ophthalmology, King's College London, St. Thomas’ Hospital Campus, London, United Kingdom. E-mail: o.mahroo@ucl.ac.uk.

The study by Panneman et al.^1^ describes in detail three cases of early-onset severe retinal dystrophy in association with *ABCA4* variants. They thus demonstrate the potential extremes in severity in *ABCA4*-retinopathy, likely influenced by disease modifiers, and reports such as these highlight the importance of efforts to identify potential modifiers in inherited retinal disease. The authors consider whether the variants they identified in other genes might act as modifiers, and the possibility of further, unidentified, genetic and non-genetic modifiers.

In the context of *ABCA4*-retinopathy, an important potential environmental modifier could relate to intake of vitamin A. Vitamin A supplementation has been shown to exacerbate lipofuscin accumulation in mouse models of the disease,^2^ and such studies have informed the development of experimental therapies that aim to modulate or alter retinal vitamin A processing with the aim of ameliorating disease.^3^ In the context of the three patients reported by Panneman et al., it would be informative (for understanding the potential mechanisms underlying the phenotypic severity) to explore whether they had undergone vitamin A supplementation at an early age. Some countries introduced high dose vitamin A supplementation to children aged 6 months to 5 years to combat high levels of infant vitamin A deficiency.^4^ India instituted such a program from 1970.^5^ Two of the three patients were from India and would have been born during the period covered by this program.

The other patient was from the United States where vitamin A deficiency is not such a major public health problem. However, in the first half of the 20th century, there were several intervention studies exploring high doses of vitamin A, including in infants, and vitamin A supplementation was also being promoted in industry advertisements targeted at the general public.^6^ This patient was the oldest of the three (he was aged 43 years in 1990). At the time of his birth (which would have been shortly after the second world war), it is possible that high dose vitamin A supplementation was occurring in parts of the United States.
